# Further slowing down of hydrolysis of amylose heated with black soybean extract by treating with nitrite under gastric conditions

**DOI:** 10.1038/s41598-022-17476-6

**Published:** 2022-08-02

**Authors:** Umeo Takahama, Sachiko Hirota

**Affiliations:** 1 Emeritus Professor of Dentistry, Kyushu Dental University, Kitakyushu, 803-8580 Japan; 2 Sanyo-Gakuen College, Okayama, 703-8501 Japan

**Keywords:** Carbohydrates, Enzymes

## Abstract

Black soybean (BSB), which contains cyanidin-3-*O*-glucoside (C3G) and procyanidins, is cooked with rice in Japan. The color of the cooked rice is purplish red due to the binding of C3G and reddish oxidation products of procyanidins. These components can slowdown pancreatin-induced hydrolysis of amylose more significantly than the hydrolysis of amylopectin, and can react with nitrous acid in the stomach. This manuscript deals with the effects of nitrous acid on pancreatin-induced hydrolysis of amylose heated with BSB extract. The hydrolysis of amylose heated with BSB extract was slow, and the slowdown was due to the binding of C3G/its degradation products and degradation products of procyanidins. The amylose hydrolysis was slowed down further by treating with nitrite under gastric conditions. The further slowdown was discussed to be due to the binding of the products, which were formed by the reaction of procyanidins with nitrous acid, to amylose. In the products, dinitroprocyanidins were included. In this way, the digestibility of amylose heated with BSB extract can be slowed down further by reacting with nitrous acid in the stomach.

## Introduction

Yellow soybean (*Glycine max* (L.) Merr.) is an important food in the world as a source of proteins. In addition, the soybean is used as a source of isoflavones that are beneficial for human health. In the varieties, black soybean (BSB) is included. This soybean accumulates anthocyanins in the seed coat. The major anthocyanin is cyanidin 3-*O*-glucoside (C3G)^1–3^. In addition, procyanidins including procyanidin B2 (proB2) are accumulated in the seed coat^4–7^.

In Japan, BSB is cooked with rice. By the cooking, the flavonoids including C3G bind to rice turning its color into purplish red. Flavonoids including anthocyanidins have been reported to be able to interact with starch non-covalently inhibiting its digestion^8–11^. In addition, it has been reported that the slow starch digestion of rice cooked with adzuki bean may be due to the binding of the degradation products of procyanidin to amylose^12,13^. Furthermore, it has been reported on the contribution of procyanidins to slow down amylose hydrolysis of non-glutinous rice flour heated with BSB extract^14^ and on the mechanism of slowdown of amylose hydrolysis in corn starch and potato starch heated with procyanidin C1^15^. Taking the reports into account, we postulated that the hydrolysis of a reagent amylose might be slowed down by heating with BSB extract.

Nitrate ingested as a food component is absorbed into the body, and then secreted into the oral cavity as a salivary component. The secreted nitrate is reduced to nitrite by the nitrate reducing bacteria^16^. The nitrite produced in the oral cavity can be mixed with foods, and then the mixture was swallowed. The swallowed nitrite is protonated in the stomach (pH, approximately 2) forming nitrous acid (p*Ka* = 3.3), which can react with food components such as flavonoids producing nitric oxide (NO)^17^. It has been reported that nitrous acid can decrease quercetin-dependent inhibition of starch digestion^18^, and does not affect the starch digestion of rice cooked with adzuki bean^13^.

As described above, pancreatin-induced hydrolysis of amylose is more effectively inhibited by flavonoids including procyanidins than that of amylopectin^12–14^. This manuscript deals with the effects of BSB extract, C3G, and proB2 on pancreatin-induced hydrolysis of a reagent amylose at first, and then the effects of nitrous acid on the hydrolysis of amylose heated with BSB extract, C3G, and proB2. Taking the results obtained in this study into account, mechanisms of the inhibition of amylose hydrolysis before and after treating with nitrous acid are discussed, and it is proposed that slowly hydrolysable starch can be prepared by cooking amylose-containing starch with procyanidin-rich foodstuffs.

## Results and discussion

### Effects of BSB extract, C3G, and proB2 on amylose hydrolysis

#### Effects of BSB extract

**Figure 1:**
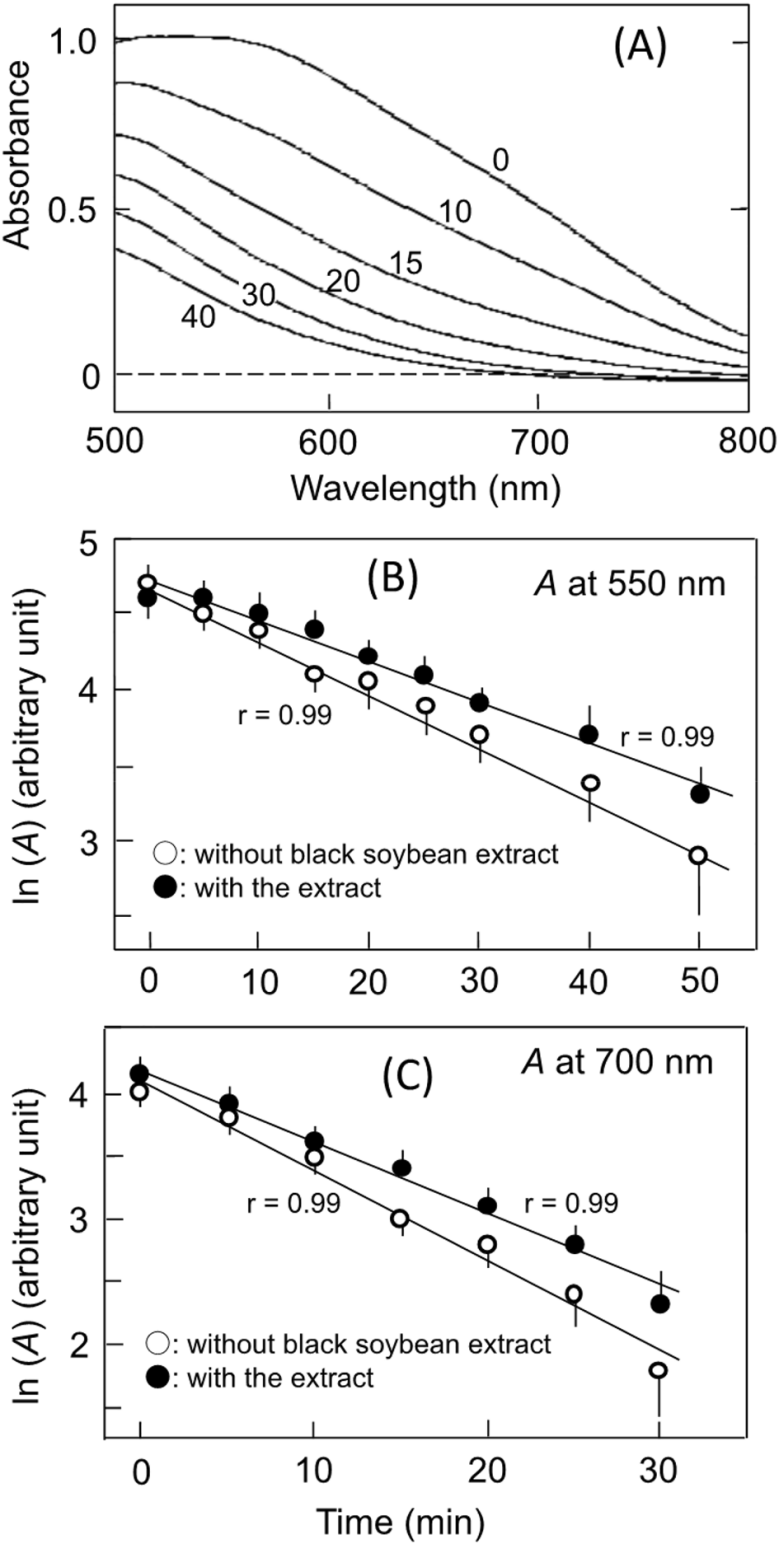
Pancreatin-induced hydrolysis of amylose heated with and without BSB extract. (**A**) Hydrolysis of amylose heated without BSB extract. Numbers on traces; incubation time in min. (**B**) and (**C**) Semi-logarithmic plots of absorbance decrease at 550 and 700 nm, respectively. (○) Heated without BSB extract, (●) heated with BSB extract. Each data point is mean with standard deviation (n = 3). *p < 0.05. The straight lines were estimated using a least squares method.

Figure 1A shows pancreatin-induced hydrolysis of amylose heated without BSB extract. Before the addition of pancreatin (trace 0), amylose-iodine complexes had a broad peak around 550 nm and a significant absorbance around 700 nm. Accompanying the hydrolysis, the absorbances decreased shifting the peak to shorter wavelengths. The absorbance decreases at 550 and 700 nm were nearly linear as a function of incubation time when plotted semi-logarithmically (Fig. 1B,C), suggesting that the hydrolysis was a first-order like reaction. The hydrolysis of amylose heated with the BSB extract prepared as described in section "Effects of BSB extract, C3G, and proB2 on starch hydrolysis" was also a first-order like reaction (Fig. 1B,C). The half-life at 550 and 700 nm was increased significantly by the extract, and that the increase was accompanied by the inhibition of reducing sugar formation (Table 1). The half-life of the hydrolysis of amylopectin seemed not to be affected significantly by heating with BSB extract (data not shown).

**Table 1:** Effects of BSB extract, C3G, and proB2 on pancreatin-induced hydrolysis of amylose.

Additions	Half-lives (min)^a^						Reducing sugar formation (relative to heated without additions)		
	550 nm			700 nm					
	Heated without	Heated with	After heating	Heated without	Heated with	After heating	Heated without (%)	Heated with	After heating
BSB extract^b^	20.6 ± 1.1	26.0 ± 2.8^c^	NE^d^	7.8 ± 0.9	10.8 ± 1.4^c^	NE^d^	100	62 ± 6%	NE^d^
0.2 mM C3G	19.7 ± 2.1	23.4 ± 1.1^c^	26.3 ± 3.8^c^	7.7 ± 0.4	9.4 ± 1.1^c^	9.3 ± 0.8^c^	100	83 ± 2%	74 ± 3%
0.2 mM proB2	20.9 ± 1.8	24.9 ± 2.2^c^	21.1 ± 1.4	9.8 ± 0.5	12.0 ± 0.3^c^	9.2 ± 0.6	100	82 ± 4%	96 ± 1%

^a^The values were determined as Fig. 1

^b^Concentration of C3G in BSB extract, 0.2 mM.

^c^Significant difference between “Heated without” and “Heated with”, and between “Heated without” and “After heating”. *p* < 0.05 (n = 3).

^d^Not examined.

#### Effects of C3G

Amylose was also hydrolyzed by a first-order like reaction as Fig. 1B and C, when heated with 0.2 mM C3G or 0.2 mM C3G was added to heated amylose. The half-life was increased by C3G, and the increase was accompanied by the inhibition of reducing sugar formation (Table 1). On the other hand, C3G did not significantly affect the half-life of the hydrolysis of amylopectin when heated with and when added to heated amylopectin. The result suggests that C3G could bind to amylose inhibiting its hydrolysis, but could not bind to amylopectin. Even if bound to amylopectin, the binding was not strong enough to prevent the access of α-amylase to amylopectin. It has been reported that C3G bound to amylopectin branches diffuses out readily^19^.

When 0.2 mM C3G was added to heated amylose, the molar ratio of C3G to amylose was calculated to be 1:11 under the conditions of this study, suggesting that approximately 10% of the amylose molecules might be combined with one molecule of C3G. The data in Table 1 indicate that the binding increased the half-lives at 550 and 700 nm by about 33 and 21%, respectively, and inhibited the reducing sugar formation by about 25%, suggesting that one molecule of C3G might be able to slow down the hydrolysis of more than two amylose molecules. The slowdown might be due to C3G-dependent combining of two amylose molecules. Some flavonoids have been reported to be able to combine two amylose molecules^15,20,21^.

The concentration of C3G heated with amylose for 30 min decreased from 0.2 mM to approximately 0.06 mM, when C3G concentration was estimated by adding 2 mL of 1% HCl in methanol to 2 mL of the heated mixture. The half-lives of amylose hydrolysis at 550 and 700 nm were increased by about 19 and 22%, respectively, and reducing sugar formation was inhibited by about 17% by the heating with C3G (Table 1). These results suggest that not only C3G itself but also its degradation products could inhibit amylose hydrolysis. In this study, 3,4-dihydroxybenzoic acid was tentatively identified as one of the degradation products by HPLC. It has been reported that benzoic acids are produced from cyanidin by heating^22^, and that phenolic acids can make complexes with amylose inhibiting its digestion^23,24^.

#### Effects of ProB2

Pancreatin-induced hydrolysis of amylose, which had been heated with 0.2 mM proB2, was also a first-order like reaction as Fig. 1B and C. The half-life was increased and the reducing sugar formation was inhibited by the procyanidin (Table 1). On the other hand, when added to heated amylose, 0.2 mM proB2 had no significant effects on the half-life and the reducing sugar formation (Table 1). These results suggest that the components produced from proB2 during the heating participated in the inhibition under the conditions of this study. When heated with amylopectin and when added to heated amylopectin, 0.2 mM proB2 did not affect its hydrolysis significantly. This result suggests that neither C3G/the degradation products (section "Effects of C3G") nor proB2/the degradation products could bind strongly to the peripheral areas of amylopectin.

**Figure 2:**
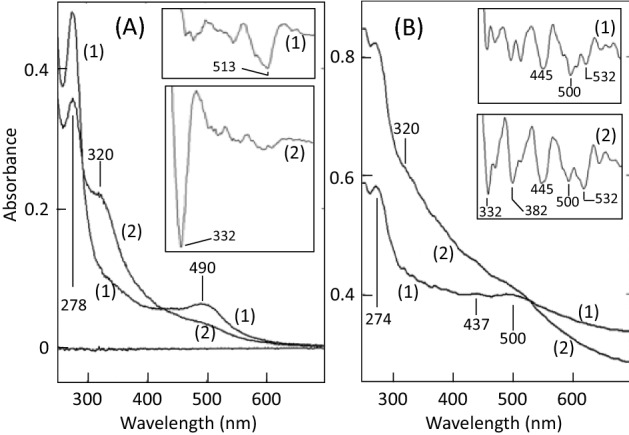
Absorption spectra of the degradation products of proB2 produced by heating and nitrous acid-treatment. Amylose was heated with 0.2 mM proB2 (section "Effects of BSB extract, C3G, and proB2 on starch hydrolysis") and the heated amylose was treated with nitrous acid (section "Effects of nitrous acid-treatment on the hydrolysis of amylose"). The supernatants and the precipitates of amylose were prepared as described in section "Absorption spectra of the degradation products of proB2". (**A**) Supernatants, (**B**) precipitates. Insets, second differential spectra. Δ*λ* = 20 nm. Traces (1), heated samples; traces (2), heated and nitrous acid-treated samples.

During the heating of the mixture of amylose and proB2, the color turned into pale red. Reddish supernatant and reddish precipitate were prepared as described in section "Absorption spectra of the degradation products of proB2". The supernatant had a peak around 490 nm (Fig. 2A, trace 1); the second differential spectrum had a negative peak at 513 nm (trace 1 in the inset). The precipitate had peaks around 437 and 500 nm (Fig. 2B, trace 1); the second differential spectrum had negative peaks at 445, 500, and 532 nm (trace 1 in the inset), suggesting that these components might contribute to the inhibition of amylose hydrolysis.

The amylose hydrolysis was not inhibited when proB2 was added to heated amylose, whereas the hydrolysis was inhibited when C3G was added to heated amylose (Table 1). The different effects might be attributed to the difference in their hydrophobicity, because the inhibition of starch digestion by some flavonoids increased with the increase in their hydrophobicity^18^. The hydrophobicity of C3G was estimated to be greater than that of proB2 from their retention times measured using a reverse phase HPLC column (proB2, 7.8 min; C3G, 11.2 min).

The above results suggest that when amylose was heated with BSB extract, C3G/its degradation products and the degradation products of procyanidins in the extract could slow down the pancreatin-induced hydrolysis of amylose.

### Nitrous acid-dependent increase in the inhibition of amylose hydrolysis

After swallowing foods mixed with salivary nitrite, starch in the foods stays in the stomach for 2–3 h. During the staying, flavonoids in foods can react with nitrous acid (p*Ka* = 3.3). It has been reported that nitrous acid concentration in the gastric cardia ranges from 0 to 0.2 mM^25^, and that the acid can react with catechins, quercetin, procyanidins, and so forth^17,26–28^. In addition, proanthocyanidin-dependent reduction of nitrous acid to nitric oxide (NO) in the stomach accompanying the ingestion of kneaded buckwheat flour is also reported^29^. Here, amylose heated with BSB extract, C3G, or proB2 was treated with 0.2 mM nitrite at pH 1.7, and then the amylose hydrolysis was studied.

The hydrolysis of amylose heated without the above extract and reagents was also a first-order like reaction as Fig. 1B and C, even after the treatment with nitrous acid. The treatment of amylose heated with BSB extract and proB2 resulted in the increase in the half-life and slowed down the reducing sugar formation, but the treatment of amylose heated with C3G did not affect the hydrolysis reactions (Table 2). These results suggest that the nitrous acid-dependent slowdown of the hydrolysis of amylose heated with BSB extract could attribute to the reaction of nitrous acid with procyanidins in the extract.

**Table 2:** Effects of nitrous acid-treatment on pancreatin-induced hydrolysis of amylose heated with BSB extract, C3G, or proB2.

	Half-lives (min) ^a^				Reducing sugar formation (rate relative to untreated)	
	550 nm		700 nm			
	Untreated	Treated with HNO_2_	Untreated	Treated with HNO_2_	Untreated (%)	Treated with HNO_2_
BSB extract^b^	23.9 ± 2.6	33.0 ± 2.6^c^	9.6 ± 1.3	13.8 ± 2.0^c^	100	72 ± 8%
0.2 mM C3G	23.6 ± 1.0	22.7 ± 3.4	10.3 ± 0.3	10.0 ± 1.0	100	101 ± 2%
0.2 mM proB2	23.2 ± 0.9	32.2 ± 3.7^c^	10.5 ± 0.4	13.5 ± 0.6^c^	100	65 ± 9%

^a^The values were determined as Fig. 1

^b^Concentration of C3G in BSB extract, 0.2 mM.

^c^Significant difference between “Untreated” and “Treated with HNO_2_”. *p* < 0.05 (n = 3–4).

The pale red color of amylose heated with proB2 was turned into pale yellow by the decrease in pH from 7.0 to 1.7, and the color changed to pale brown by treating the heated amylose/proB2 with nitrous acid. The supernatant prepared from the nitrous acid-treated amylose/proB2 (section "Absorption spectra of the degradation products of proB2") did not have distinct absorption bands in the visible wavelength region, but had a shoulder around 320 nm (Fig. 2A, trace 2); the second differential spectrum had a negative peak at 332 nm (trace 2 in the inset). The absorption band could be attributed to dinitrosoproB2. It has been reported that the compound is formed in proB2/nitrous acid systems^26–28^ and has a peak around 328 nm in the mixture of methanol and 25 mM KH_2_PO_4_ (2:7, v/v)^27^.

The precipitate had several shoulders (Fig. 2B, trace 2); the second differential spectrum showed that nitrous acid induced the formation of components with negative peaks at 332 and 382 nm (compare trace 1 and trace 2 in the inset). The two components might contribute to the nitrous acid-dependent slowing down of amylose hydrolysis. The component with the peak at 332 nm was deduced to be dinitrosoproB2 by comparing the secondary differential spectrum of trace 2 (inset) in Fig. 2B with that of trace 2 (inset) in Fig. 2A. The characteristics of the component with a peak 382 nm, which was not found in the supernatant, were unclear at present.

Although nitrous acid did not affect the hydrolysis of amylose heated with C3G (Table 2), C3G concentration in heated amylose was decreased by about 40% by the nitrous acid when estimated spectrophotometrically (section "Effects of C3G"). The ineffectiveness might be due to the binding of the products, which were formed by the reaction of nitrous acid with C3G/its degradation products, to amylose in the similar way as C3G/its degradation products.

Taking the above result and the presence of procyanidins including proB2 in BSB seed coat into account (see Introduction), it could be postulated that dinitrosoprocyanidins and their oxidation products could contribute to the nitrous acid-induced slowdown of the hydrolysis of amylose heated with BSB extract. Further studies are required to elucidate the chemical structures of the components formed from procyanidins accompanying the heating and the nitrous acid-treatment.

## Conclusion

It became clear form the present study that pancreatin-induced amylose hydrolysis was slowed down by heating with BSB extract, C3G, and proB2. As the mechanism of the inhibition by BSB extract, binding of C3G/its degradation products and the degradation products of procyanidins including proB2 to amylose was proposed. The inhibitory effect of BSB extract increased further by nitrous acid-treatment, and the inhibitory effects of proB2 but not C3G was increased further by nitrous acid-treatment. Taking the results into account, nitrous acid-dependent increase in the inhibitory effects of BSB extract could be attributed to the binding of dinitrosoprocyanidins and the oxidation products to amylose. The above results suggest that foods, the amylose hydrolysis of which is slow, can be produced by heating amylose-containing foods with procyanidin-rich foodstuffs, and that amylose hydrolysis in the intestine can be slowed down if amylose/procyanidin complexes react with nitrous acid in the stomach.

## Materials and methods

### Ingredients and reagents

Black soybean (BSB) (*Glycine max* (L.) Merr.) produced in Hokkaido was obtained from a local market. 3,4-Dihydroxybenzoic acid, 4-hydroxybenzhyrazide, and pancreatin from porcine pancreas were from FUJIFILM Wako (Osaka, Japan). According to their data, activities of digestive enzymes in the pancreatin are following: protease (26 − 46 units mg^−1^), α-amylase (3 − 5 units mg^−1^), lipase (0.75 − 1.4 units mg^−1^). C3G was obtained from Nagara Science (Gifu, Japan), and proB2 was from Funakoshi (Tokyo, Japan). A reagent amylose (synthetic) (BAR-5 K-1) (average molecular weight, 4500) was from PS-Biotec Inc. (Osaka, Japan) and a reagent amylopectin from waxy corn was from Tokyo Chemical Industry (Tokyo, Japan). Iodine solution (100 mM) was prepared as described previously^12^.

### Measurement of absorption spectra and HPLC analysis

Absorption spectra were measured using a spectrophotometer (UV-2450) equipped with an integrating sphere assembly (ISR-240A) (Shimadzu, Kyoto, Japan). Path length of the measuring beam was 4 mm.

HPLC to identify C3G and its degradation products was performed at 40 °C using two pumps CL-10AD, a system controller SCL-10Avp, a reverse phase column VP-ODS (15 cm × 4.6 mm inner diameter), and a spectrophotometric detector with a photodiode array SPD-M10Avp (Shimadzu, Kyoto, Japan). The mobile phase was consisted of methanol and 0.2% formic acid. Its methanol concentration was kept 0% during the initial 5 min after the injection of samples, and then the methanol concentration was increased linearly to 100% in 30 min. The difference in the hydrophobicity between C3G and proB2 was estimated using 40% methanol in 0.2% formic acid. Flow rate of the mobile phases was 1 mL min^−1^.

### Effects of BSB extract, C3G, and proB2 on starch hydrolysis

Seed coat of BSB (0.15 g) was ground in 5 mL of 0.1 M sodium phosphate (pH 7.0) with 0.15 M NaCl using a mortar and a pestle. Methanol (5 mL) was added to the ground seed coat, and centrifuged at 1830* g* for 5 min. The anthocyan in the supernatant was identified to be C3G by comparing the retention time and absorption spectrum with standard C3G using the above HPLC system. Its concentration was estimated from the absorbance at 520 nm of the mixture of the supernatant (0.1 mL) and 1% HCl in methanol (1 mL). The absorbance coefficient used was 27 mM^−1^ cm^−1^^30^. The concentration of proB2 in the supernatant was not determined in this study, but the total concentration of procyanidin dimers could be estimated using the amount of C3G and the total amount of procyanidin dimers presented in Table 1 of ref. 7. The concentration was approximately half of the concentration of C3G.

The concentration of C3G in the supernatant of BSB extract was adjusted to 0.2 mM by adding 0.1 M sodium phosphate (pH 7.0) with 0.15 M NaCl. Amylose or amylopectin (20 mg) was added to the supernatant or the above buffer solution (2 mL), and then heated in gently boiling water for 30 min. During the heating, the starch suspensions became transparent. The heated amylose or amylopectin was left for 15 min at room temperature, and the volume was adjusted to 2 mL with water to preincubate for 10 min at 37 °C. Starch hydrolysis was initiated by adding pancreatin (10 µg mL^−1^) to the preincubated reaction mixtures. After adding pancreatin, an aliquot (20 µL) of the reaction mixture was withdrawn at predetermined time intervals to measure the starch hydrolysis and reducing sugar formation as described in section "Measurements of starch hydrolysis".

In addition, amylose or amylopectin (20 mg) suspended in 2 mL of 0.1 M sodium phosphate (pH 7.0) with 0.15 M NaCl with and without 0.2 mM C3G or proB2 was heated in gently boiling water for 30 min. These mixtures were used as “heated with and without additions”. On the other hand, the above reagents were added to amylose and amylopectin, which had been heated for 30 min in the above buffer solution, and then left for 15 min at room temperature. The mixtures were used as “added after heating”. After adjusting the volume to 2 mL with water, the above mixtures were preincubated for 10 min at 37 °C to initiate starch hydrolysis by adding pancreatin (10 µg mL^−1^). After the addition of pancreatin, an aliquot (20 µL) of the reaction mixture was withdrawn to measure the starch hydrolysis and reducing sugar formation as described in section "Measurements of starch hydrolysis".

### Measurements of starch hydrolysis

An aliquot (20 µL) withdrawn as described above (section "Effects of BSB extract, C3G, and proB2 on starch hydrolysis") and 0.1 mL of 100 mM iodine solution were added successionally to 1 mL of 0.1 M sodium phosphate (pH 7.0) with 0.15 M NaCl. The mixture was left for 10 min at room temperature to measure the absorption spectrum.

Reducing sugar formation was estimated as reported previously^31^. The aliquot (20 µ L) and 0.15 mL of 0.33 M 4-hydroxybenzhyrazide dissolved in 0.6 M HCl were added successionally to 1.35 mL of a solution that contained 0.042 M sodium citrate, 0.007 M calcium chloride, and 0.5 M sodium hydroxide. The mixture was heated in boiling water for 6 min, and then centrifuged at 1830* g* for 5 min to measure the absorbance of the supernatant at 410 nm. The increase in the absorbance was nearly linear as a function of incubation time for 50 min. The rates were estimated using the least-squares method.

### Effects of nitrous acid-treatment on the hydrolysis of amylose

Amylose (20 mg) was heated with 2 mL of BSB extract equivalent to 0.2 mM C3G, or heated with 0.2 mM C3G or proB2 dissolved in 2 mL of 0.1 M sodium phosphate (pH 7.0) with 0.15 M NaCl as described in section "Effects of BSB extract, C3G, and proB2 on starch hydrolysis". The pH of the heated samples was adjusted to 1.7 by 2 M HCl, and then the acidic samples were incubated with and without 0.2 mM NaNO_2_ at 37 °C for 30 min. After the incubation, the acidic samples were neutralized with saturated Na_2_HPO_4_. The amylose hydrolysis was initiated as described in section "Effects of BSB extract, C3G, and proB2 on starch hydrolysis", and the hydrolysis was estimated as described in section "Measurements of starch hydrolysis". When the reducing sugar formation was estimated, the concentration of calcium chloride was 0.017 M.

### Absorption spectra of the degradation products of proB2

Amylose suspensions, which had been heated with 0.2 mM proB2, were incubated for 30 min with and without 0.2 mM nitrous acid at pH 1.7, and then neutralized (section "Effects of nitrous acid-treatment on the hydrolysis of amylose"). The neutralized suspensions were kept at 4 °C for one day. Amylose precipitates generated during the keeping were collected by centrifugation at 1830 g for 10 min. The absorption spectra of the supernatants and the precipitates, which were suspended in 2 mL of 0.1 M sodium phosphate (pH 7.0) with 0.15 M NaCl, were measured to characterize the components bound to amylose.

### Statistical analysis

All experiments were repeated more than three times. Data were presented as averages with standard deviations. Significant difference between two samples was determined using Student’s *t*-test, setting the significance threshold to 0.05 (student’s t-test https://keisan.casio.jp).

## Data Availability

The datasets obtained during the current study are available from the corresponding author on reasonable request, and materials can be obtained commercially.

## Abbreviations

BSB: Black soybean
C3G: Cyanidin 3-*O*-glucoside
proB2: Procyanidin B2
